# Cost-efficient high-throughput HLA typing by MiSeq amplicon sequencing

**DOI:** 10.1186/1471-2164-15-63

**Published:** 2014-01-24

**Authors:** Vinzenz Lange, Irina Böhme, Jan Hofmann, Kathrin Lang, Jürgen Sauter, Bianca Schöne, Patrick Paul, Viviane Albrecht, Johanna M Andreas, Daniel M Baier, Jochen Nething, Ulf Ehninger, Carmen Schwarzelt, Julia Pingel, Gerhard Ehninger, Alexander H Schmidt

**Affiliations:** 1 DKMS Life Science Lab, Fiedlerstrasse 34, 01307 Dresden, Germany; 2 DKMS German Bone Marrow Center, Kressbach 1, 72072 Tübingen, Germany; 3 molpe Softwareentwicklungs GmbH, 72138 Kirchentellinsfurt, Germany; 4 Medizinische Klinik und Poliklinik I, Universitätsklinikum Carl Gustav Carus der Technischen Universität Dresden, Fetscherstrasse 74, 01307 Dresden, Germany

**Keywords:** Human leukocyte antigen, HLA typing, NGS, Dual indexing, 4-primer approach, Amplicon-based sequencing, Fluidigm Access Array, Illumina MiSeq

## Abstract

**Background:**

A close match of the HLA alleles between donor and recipient is an important prerequisite for successful unrelated hematopoietic stem cell transplantation. To increase the chances of finding an unrelated donor, registries recruit many hundred thousands of volunteers each year. Many registries with limited resources have had to find a trade-off between cost and resolution and extent of typing for newly recruited donors in the past. Therefore, we have taken advantage of recent improvements in NGS to develop a workflow for low-cost, high-resolution HLA typing.

**Results:**

We have established a straightforward three-step workflow for high-throughput HLA typing: Exons 2 and 3 of HLA-A, -B, -C, -DRB1, -DQB1 and -DPB1 are amplified by PCR on Fluidigm Access Array microfluidic chips. Illumina sequencing adapters and sample specific tags are directly incorporated during PCR. Upon pooling and cleanup, 384 samples are sequenced in a single Illumina MiSeq run. We developed “neXtype” for streamlined data analysis and HLA allele assignment. The workflow was validated with 1140 samples typed at 6 loci. All neXtype results were concordant with the Sanger sequences, demonstrating error-free typing of more than 6000 HLA loci. Current capacity in routine operation is 12,000 samples per week.

**Conclusions:**

The workflow presented proved to be a cost-efficient alternative to Sanger sequencing for high-throughput HLA typing. Despite the focus on cost efficiency, resolution exceeds the current standards of Sanger typing for donor registration.

## Background

The success of unrelated hematopoietic stem cell transplantation is closely linked to a good match of the HLA genes between donor and recipient [1]. To overcome the odds of finding a matching donor – given the high diversity of the HLA system (>8000 described alleles) – many organizations worldwide endeavor to characterize the HLA types of volunteers. In 2012 alone, 1.6 million new potential donors were registered. Typing potential donors upfront for 5 loci at high resolution would be highly beneficial in speeding up the search process, thereby saving precious time for patients in need of a transplantation [2]. However, due to the cost of high resolution HLA typing based on Sanger sequencing, many organizations restrict the number of loci typed to HLA-A,-B and-DRB1 or even restrict the number of potential donors to be recruited. After revolutionizing genome sequencing, NGS is expected to also markedly change the diagnostic market. HLA typing by NGS has been demonstrated by performing long range PCR in combination with shotgun sequencing [3-5]. However, the required sophisticated sample preparation steps and associated costs make that approach currently more attractive for patient samples than for cost-sensitive high-throughput stem cell donor registry typing. Several groups have demonstrated the feasibility of HLA typing by short amplicon sequencing on the 454 system [6,7]. Moonsamy et al. streamlined the workflow considerably by taking advantage of the Fluidigm Access Array technology for performing up to 48 × 48 (2048) PCR reactions on a single chip [8]. Given the improvements in read length to 2 × 250 base pairs, the Illumina MiSeq system seemed compatible for an amplicon sequencing approach as well. We have therefore set out to transfer the strategy of Moonsamy et al. to the MiSeq. In contrast to the 454 system, the MiSeq features on-board clonal amplification, thereby greatly simplifying sample preparation and rendering the workflow more amendable for high-throughput routine operation. In addition, the operational cost is greatly reduced, with a more than 50 fold reduced cost per read compared with the 454 GS FLX system. Taken together, those features allowed us to develop a high-throughput low-cost HLA sequencing workflow that would render typing of the transplantation-relevant genes and exons affordable for donor centers.

## Results and discussion

### Workflow

**Figure 1:**
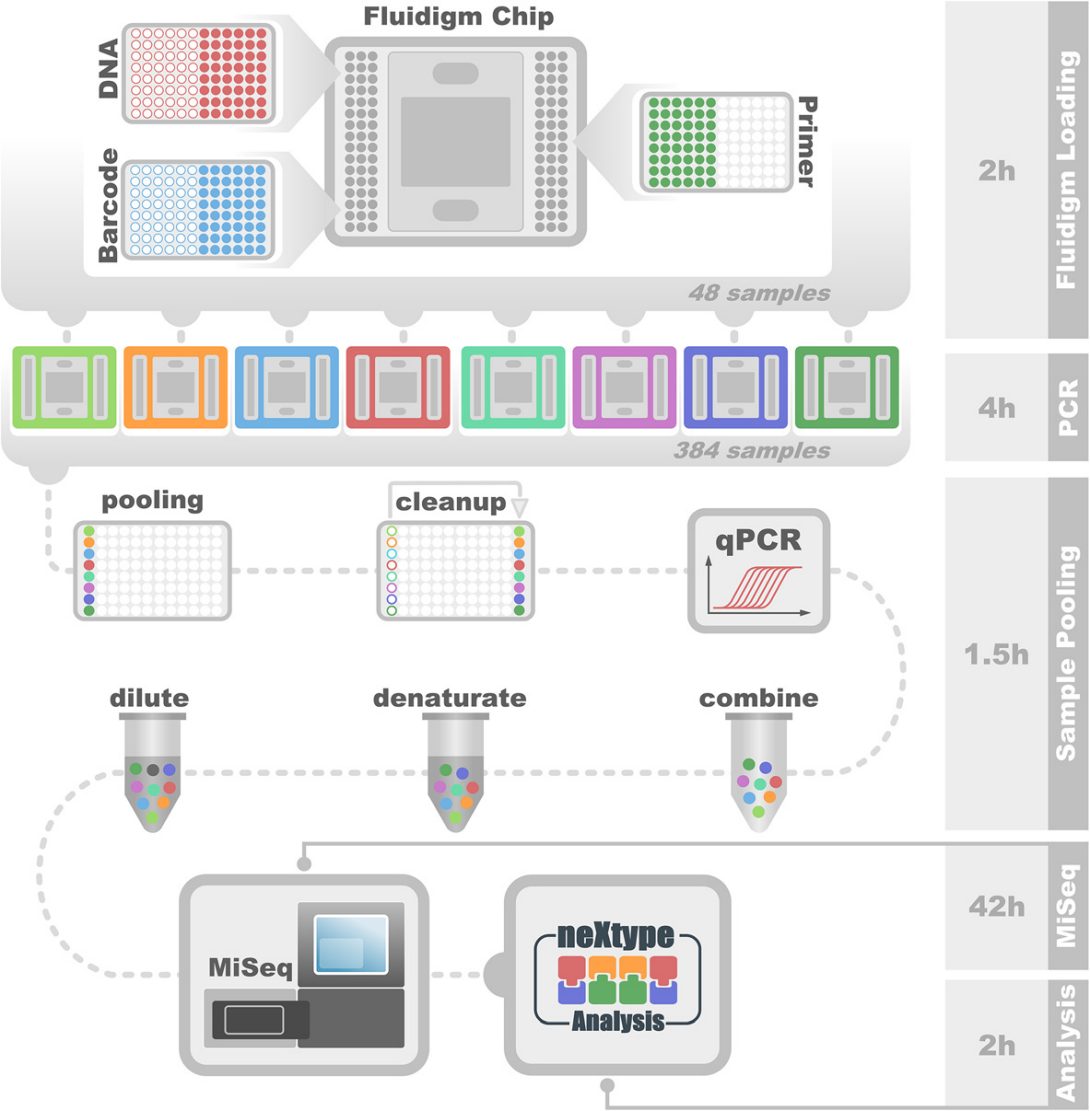
**Workflow for analyzing 384 samples.** 8 Fluidigm chips with 48 samples each are pooled for one MiSeq run.

**Figure 2:**
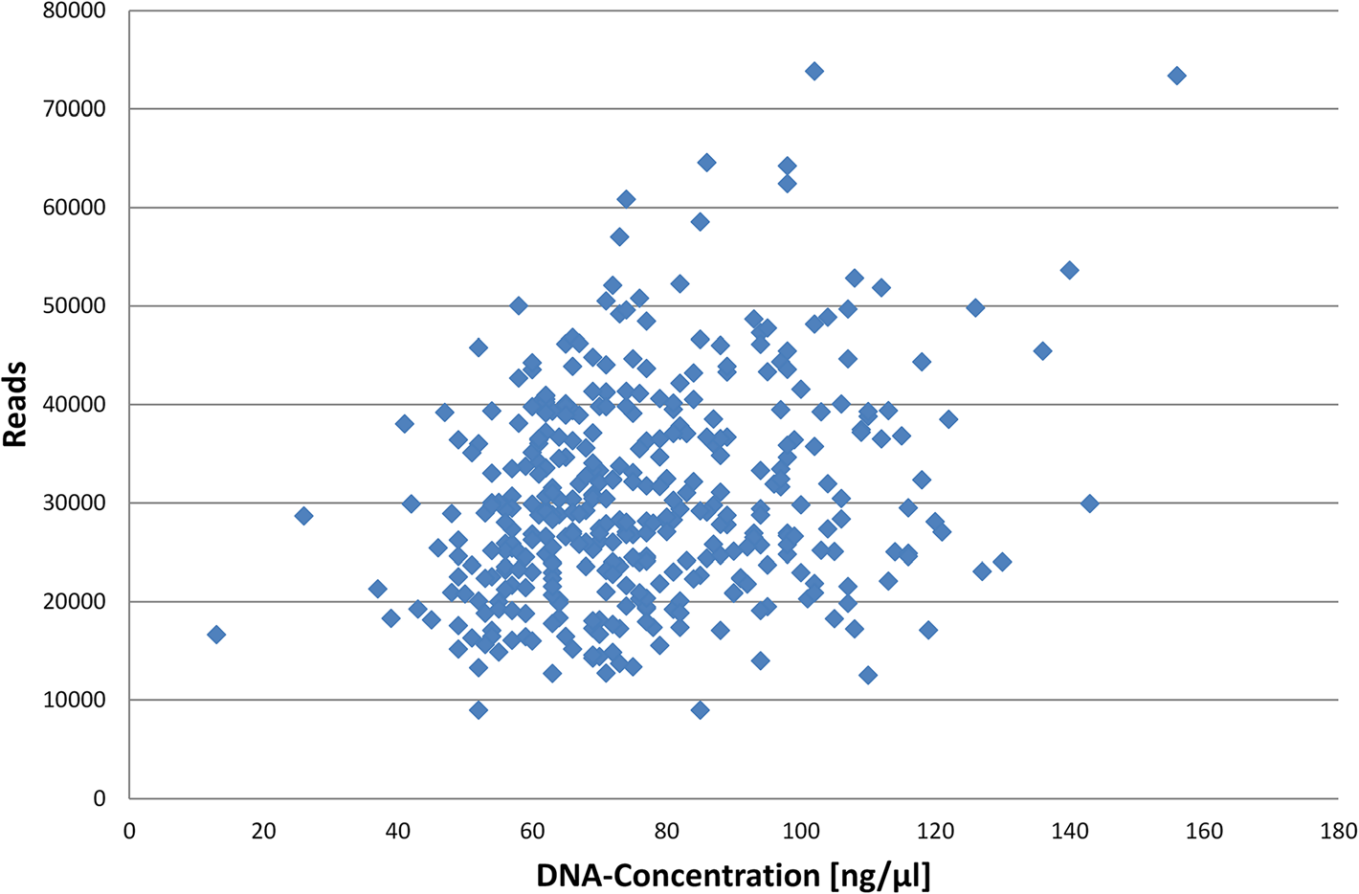
**Correlation of DNA concentration and total reads for 384 samples.** Mean DNA concentration: 77 ng/μl, mean number of reads: 30,605, coefficient of correlation: 0.26.

**Figure 3:**
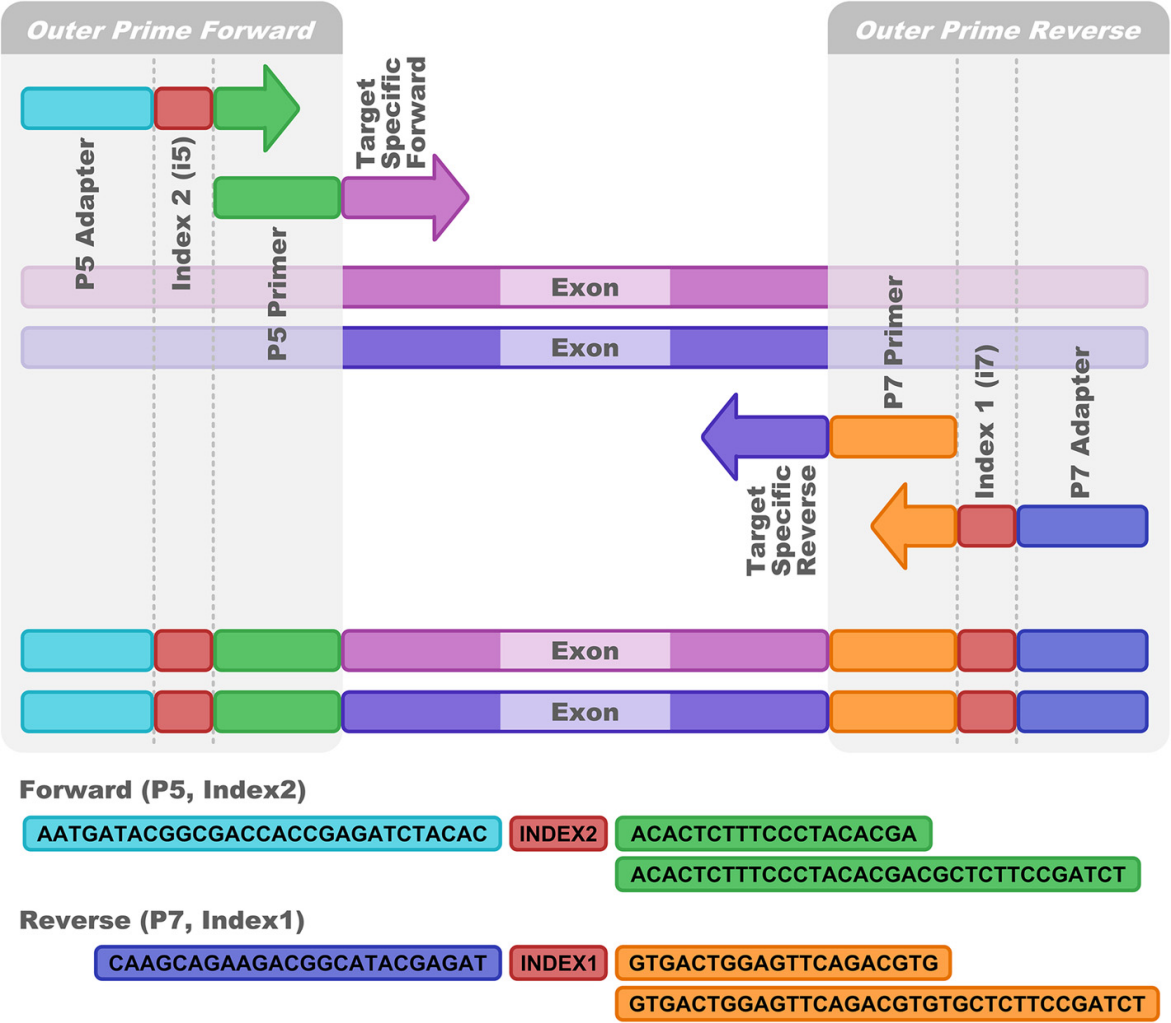
**Dual indexing in a 4-primer approach.** The 2 outer and 2 inner primers are combined in one PCR reaction to yield a MiSeq compatible product with dual indexing. Each sample is first mixed with a unique combination of outer primer indexes. The 48 samples are then combined with up to 48 target-specific primer sets in 2304 separate reaction chambers on the Fluidigm chip for PCR.

The workflow (Figure 1) consists of three straightforward steps starting with DNA isolated from human blood. Since we did not find a correlation between the number of reads and pre-PCR DNA concentration (Figure 2), we have omitted DNA normalization for the sake of cost and simplicity. HLA-specific DNA amplification by PCR is performed on Fluidigm Access Array microfluidic chips [8]. These chips feature the combination of 48 samples with 48 primer groups for PCR amplification in 2048 individual 35 nl reaction chambers. Before application to the Fluidigm chip, the 48 samples are mixed with primer sets containing unique indexing nucleotide sequences and adapter sequences to allow direct sequencing of the PCR products on the MiSeq without the need of additional library preparation steps (Figure 3). We take advantage of the capabilities of the MiSeq to sequence two indices in addition to the two paired-end 250 base pair sequencing reads. Using 8 bases for each index, we employed 96 index sequences for index1 and 16 index sequences for index2, resulting in 1536 unique combinations. During each MiSeq run, we multiplex 384 samples. Therefore, 3 out of 4 index combinations are not used in a particular run, minimizing index mis-assignments [9]. Following PCR, the 48 samples of one Fluidigm chip are pooled. The pool is purified using solid phase reversible immobilization (SPRI) bead technology with a lower DNA size cut-off of 250 base pairs to remove primer-dimer products. The purified pool is quantified by qPCR to adjust for slightly different amplification efficiencies of individual chips. Aliquots of 8 purified pools corresponding to 384 samples are combined, denatured, diluted and loaded together with a spike of 10% PhiX onto a MiSeq instrument.

### Optimization of primers and PCR conditions

**Figure 4:**
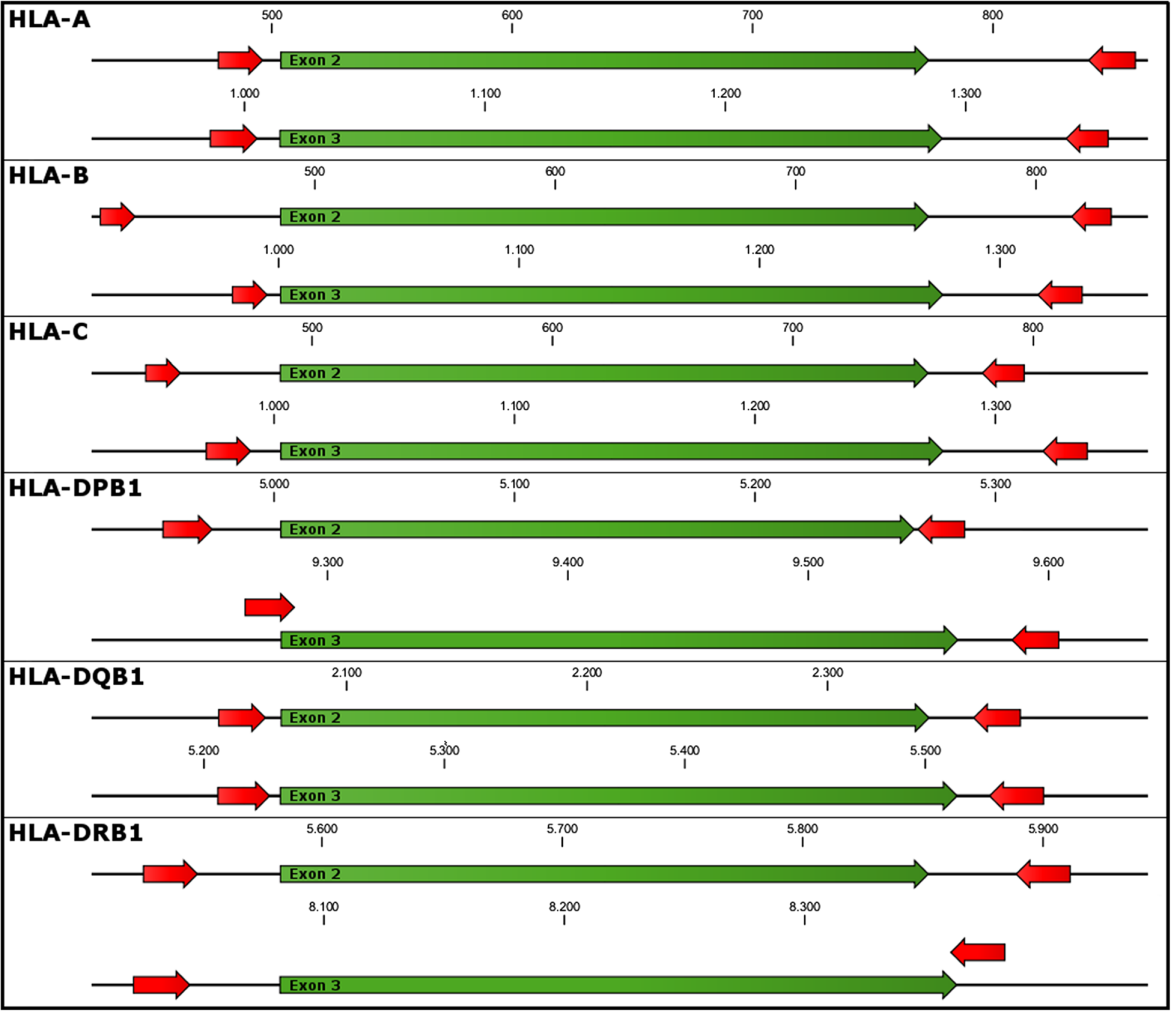
**Location of HLA specific primers.** Primers are located in the introns surrounding exon 2 or 3 respectively. There is no overlap with the exonic sequence with the exception of HLA-DQB1 exon 3 forward and HLA-DRB1 exon 3 reverse which overlap by few bases with the exonic sequence.

The primer sets are key to a successful short amplicon HLA typing strategy. We developed primer sets targeting HLA-A, -B, -C, -DRB1, -DQB1 and -DPB1, restricting ourselves to exons 2 and 3 for all loci (Figure 4). Exons 2 and 3 (of HLA class I genes; only exon 2 for HLA class II genes) represent the antigen recognition site and are therefore regarded as most relevant for unrelated stem cell transplantation [10-12]. However, limiting the sequenced region to exon 2 and 3 is a compromise which leaves unresolved certain allele ambiguities that are outside this region. Since the medical relevance of such ambiguities (with the exception of null alleles) is uncertain, they are currently not considered for donor selection in the context of unrelated stem cell transplantation. Therefore, in the context of registry typing, to focus on exon 2 and 3 is justifiable.

**Figure 5:**
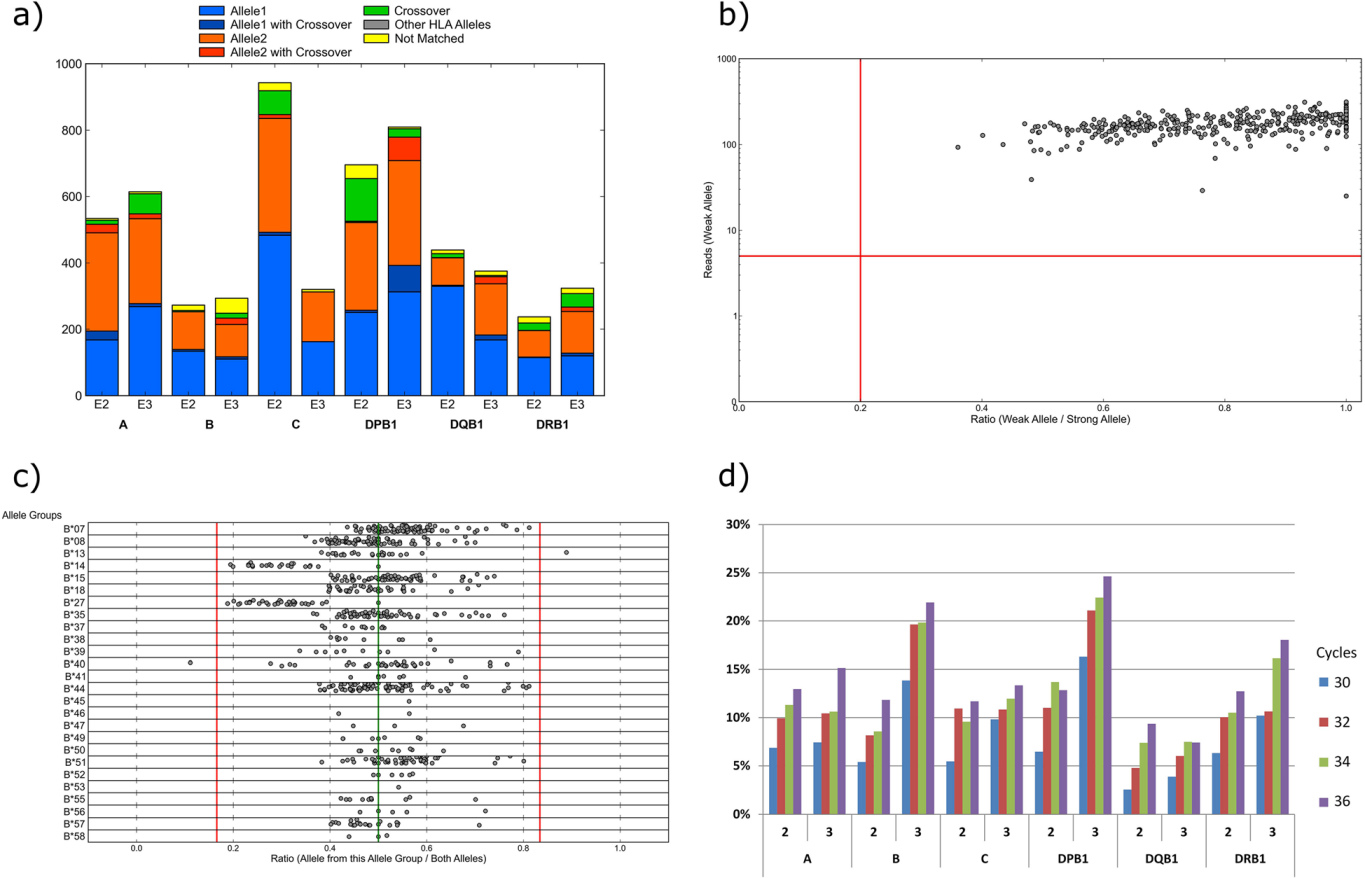
**Optimization and performance of primer sets. (a)** Classification of reads based on known typing results using sequence and Q-values. PCR artifacts resulting in artificial hybrids of allele1 and allele2 are reported as “crossover“. **(b)** Optimization of primer sets - Allele balancing: Example of an optimized primer set (A Exon 2) demonstrating balanced amplification and sufficient read counts. **(c)** Optimization of primer sets - Allele amplification bias: Example of an unoptimized primer set (B Exon 3) demonstrating negative amplification bias for allele groups B*14 and B*27. **(d)** Crossover artifact quantification: 48 samples were amplified using 30 to 36 PCR cycles and the rate of crossover formation was quantified for each locus and exon. Sample-loci with homozygous results were not considered for analysis. Lowering the number of PRC cycles reduces the crossover-rate.

HLA typing poses a particular challenge for primer selection. Despite the enormous diversity, well-balanced amplification of both alleles – independent of the allele combination – needs to be assured. High specificity is required to distinguish several evolutionary related, highly homologous genes. Furthermore, to cover the full exons of around 270 base pairs, the primers may not be too distant from the exon-intron boundaries. We developed a rigorous procedure based on the rich content of NGS data for testing the primer sets for specificity and even amplification using cohorts of 96 samples with known HLA type (Figure 5). This allowed us to fine tune the primer sets for optimal performance:

In contrast to Sanger based sequencing, using NGS unspecific amplification does not result in noisy sequencing data. Due to the clonal sequencing, the unspecific amplification products are easily identified by the analysis software. Nevertheless, specificity is preferred to maximize the relevant information of the available reads.

Likewise, with regard to unbalanced amplification, the NGS approach is considerably more sensitive in detecting suppressed alleles than Sanger sequencing which requires at least 5% to 20% of the weaker allele for detection [13]. In contrast, given sufficient read depth, our analysis software “neXtype” identifies suppressed alleles down to 2% read count relative to the dominant allele. In addition, to avoid false homozygous results, neXtype blocks homozygous calls with less than 100 reads (20 fold above the detection limit). Despite the increased sensitivity for suppressed alleles, we went through several rounds of primer optimization to come up with a set of primers with well-balanced amplification independent of the given allele combination (Figure 5 b,c). As part of our quality control, every new batch of primers is tested against a set of 95 samples to confirm well-balanced amplification, i.e. no second allele detected with less than 20% of the reads of the dominant allele. This set of samples was carefully selected to represent at least 2 samples for each allele group targeted by a particular primer in the primer group.

We also monitor the occurrence of artifacts previously reported in the literature: The generation of artificial “recombinant” or “chimeric” PCR products from the two alleles present in a sample. Such “crossover” events have been described as potential error source of HLA-DRB1 typing, as particular crossover events of HLA-DRB1 and HLA-DRB3/4/5 are identical to named HLA-DRB1 alleles [14-16]. We quantified the rate of crossover products (Figure 5a). Depending on the targeted exon, an average of up to 25% of the matched reads were identified as crossover products (Figure 5d). While we are not aware of previous quantitative analyses in the context of HLA, our findings are nonetheless in accordance with reports in the context of 16S sequencing [17]. Since most of the crossover products are generated during the late PCR cycles, the artifact may be reduced by decreasing the number of PCR cycles [18] (Figure 5d). However, in the interest of maximum robustness with regard to samples with low DNA concentration, we have chosen not to reduce PCR cycles below 32 cycles but have rather extended neXtype to handle the artifacts properly.

### HLA allele calling

We developed the NGS HLA typing software “neXtype” to match the throughput of the workflow. NeXtype takes full advantage of Q values and high coverage to automate data analysis as far as possible without compromising accuracy. Statistical algorithms are implemented to a) identify new alleles, and b) distinguish between sequencing artifacts and closely related alleles. Currently, more than 95% of the typing assignments do not require any manual corrections. Taking advantage of the high number of reads per amplicon and the high quality of base-calls, we have chosen a set of highly conservative parameters for “autotyping”: Those typing assignments (82%) do not even need to be reviewed by a human analyst. To qualify for this category (among other restrictions), homozygous calls are accepted only if the read count is high enough to safely exclude a potentially suppressed second allele, taking imbalanced amplification of up to 20:1 into account. The 18% of typings not meeting the autotyping criteria remain for analyst review and are divided into three categories: 13% require user confirmation, 2.7% requiring user editing and 2.3% failed typings. Due to the high level of automation, analysis of 384 samples (2304 loci) can be performed in less than 3 hours by one trained analyst.

### Validation

We validated the workflow including neXtype analysis software by analyzing 1140 samples of known HLA type. All common alleles with frequency >0.001 in the German population were represented by at least 2 samples [19]. Using 24 Fluidigm Access Array chips we amplified 12 HLA targeted exons per sample and sequenced them in 3 MiSeq runs. In the case of 160 sample-loci (2.3%), data quality was insufficient and these were therefore excluded from further analysis. All alleles of the 160 sample-loci that failed were successfully typed by this approach in other samples. Therefore, these failures were classified as technical failures independent of HLA type. In routine operation those technical failures need to be repeated. Those failures do however not compromise HLA typing accuracy. Subsequent to analysis using neXtype, the results were compared with the typing results obtained by Sanger sequencing: All NGS neXtype results were concordant with the Sanger sequences, demonstrating error-free typing for more than 6000 sample-loci.

### Routine operation

**Figure 6:**
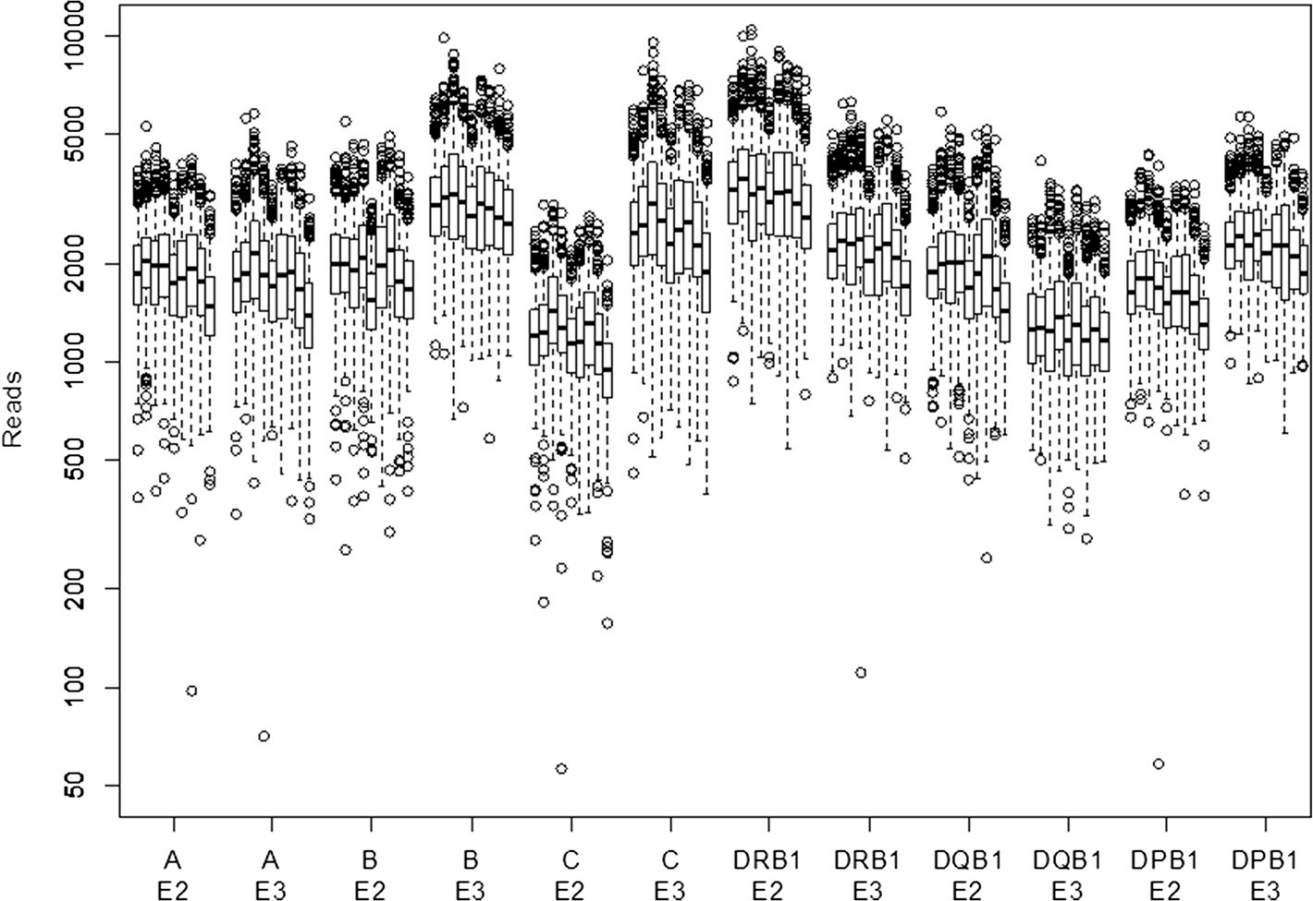
**Coverage.** Reads per amplicon and sample over 9 runs (3398 samples). Boxes represent median and first and third quartile, whiskers correspond to the interquartile range and outliers are plotted.

Upon successful validation, we started routine operations. Within 6 weeks of operations, we achieved a throughput of more than 3500 samples or 21,000 sample-loci per week. Using Sanger sequencing, more than 800 sequencing runs (96 capillaries) would be required for equal throughput. Capacity was increased to 12,000 samples per week within 12 months. The workflow has proven very reliable. Analyzing 9 early runs, only 0.6% (22 out of 3420) of the samples resulted in less than 5000 reads per sample. Depending on the targeted exon, we consistently obtain 1000 to 3000 fold coverage (median) with only 9 sample-exons (out of 20,388) below 250 reads (Figure 6).

Comparing the resolution obtained from Sanger-based registry typing with our NGS workflow demonstrates that the NGS workflow yields an increased rate of high resolution typings (Table 1). And this despite the fact that, an average of five GSSP reactions per sample were performed and analyzed on the Sanger to resolve phase ambiguities as far as possible.

**Table 1:** Resolution in comparison with Sanger based sequencing

**Locus**	**Sanger**	**NGS**
A	90.4%	99.8%
B	95.0%	97.6%
C	92.3%	95.7%
DPB1	99.8%	99.9%
DQB1	95.7%	100.0%
DRB1	96.6%	100.0%
Average	95.0%	98.8%

Rate of high resolution typing results. Resolution of 37,975 samples typed by the proposed NGS approach in September 2013 was compared with 145,932 samples analyzed by Sanger sequencing in 2012. In accordance with the standards of the European Federation for Immunogenetics (EFI), we refer to allele typing results with no ambiguities in the antigen recognition site as “high resolution”.

## Conclusion

Comparing the proposed workflow with conventional Sanger sequencing, it is apparent that the NGS workflow is more cost-efficient and easier to set up. In contrast to Sanger sequencing, the individual sample needs to be handled only when it is combined with the barcode sequence, loaded onto the Access Array chip and pooled with other samples after PCR. To exclude sample mix up by design, we have set up 2 liquid handlers with barcode reading capabilities. However, the process as such could easily be handled by manual pipetting. Therefore, it appears to be an attractive alternative to Sanger sequencing even for labs with far lower throughput. Given the combined chip and sequencing run costs of about $1000 (list price Access Array chip and MiSeq Reagent Nano Kit 500 cycles), HLA typing by NGS as proposed here seems to be more cost-efficient, starting with as few as 24 samples, even when compared with Sanger sequencing costs alone. At the same time, it was possible to further increase resolution by sequencing more exons and introns of up to 48 targeted amplicons at no significant additional cost. For the purpose of typing for HLA registries, we have restricted the number of amplicons to the exons most relevant for the donor search to routinely multiplex 384 samples. In high throughput operation, we achieved a cost reduction of more than 50% compared with cost-optimized Sanger sequencing. These realized savings lower the cost for high resolution typing of volunteer donors, thus increasing the number of registered donors with the same budget and simultaneously improving typing resolution. Both factors should facilitate and accelerate the search process and thus improve the outcome of unrelated hematopoietic stem cell transplantation.

## Methods

### Samples

All samples used were blood samples, collected in venosafe 4 ml EDTA tubes (Terumo, Tokyo, Japan). Validation samples were chosen randomly from newly recruited DKMS donors in Germany. Ethnic background is self-assigned at recruitment by country code of origin. 85% of the donors described themselves as German, 7% as Turkish, 1% as polish donors, 5% came from 33 different countries and from 2% no ethnic information is reported. Upon registration, donors sign an informed consent approving HLA typing and storage of samples for future typings related to stem cell transplantation. No ethics committee approval was obtained as sequencing-based HLA typing of potential stem cell donors is standard practice for stem cell donor centers and is covered by the donor consent form signed at recruitment. DKMS Life Science Lab is an affiliated company with DKMS German Bone Marrow Donor Center and performs HLA typing on request of DKMS.

### Sanger sequencing

All samples have been typed for HLA-A, -B, -C, -DRB1, -DQB1, and -DPB1 by Sanger sequencing using inhouse primers on a 3730xl capillary sequencer (Life Technologies, Carlsbad, USA) at the time of donor registration. These typings have been used for validation. The Sanger sequencing strategy involved amplification spanning exons 2 and 3 for HLA-A, -B, -C; two separate amplicons for exons 2 and 3 for HLA-DQB1 and -DPB1 and one amplicon for Exon 2 for HLA-DRB1. All primers were located in the introns. Sequencing was carried out separately for all exons, and group specific sequencing primers (GSSP) were used to resolve ambiguities.

### DNA isolation

DNA was isolated from 150 μl whole blood (EDTA) based on magnetic bead technology with “chemagic DNA Blood kit 150 special” (PerkinElmer, Baesweiler, Germany) using “chemagic Magnetic Separation Module I” (PerkinElmer, Baesweiler, Germany). DNA was eluted in 100 μl elution buffer (10 mM Tris–HCl pH8.0) yielding concentrations of between 35 and 100 ng/μl as determined by UV on NanoQuant plate using TECAN infinite 200Pro (Tecan, Männedorf, Switzerland) plate reader.

### PCR amplification with the access array system

PCR amplification was performed using a 48.48 Fluidigm Access Array (Fluidigm Corporation, South San Francisco, USA) in combination with the Roche High Fidelity Fast Start Kit (Roche, Basel, Switzerland).

A 10 μl PCR master mix was prepared for each sample and contained 1 μl 10x buffer mix without MgCl_2_ (Roche High Fidelity Fast Start Kit), 1.8 μl 25 mM MgCl_2_, 0.5 μl DMSO, 0.5 μl 20xAA Loading Reagent (Fluidigm Corporation, South San Francisco, USA), 0.2 μl 10 mM dNTPs each (Roche High Fidelity Fast Start Kit), 0.1 μl Fast Start High Fidelity Taq Polymerase (5 U/μl) (Roche High Fidelity Fast Start Kit) and 1.9 μl PCR grade water. 6 μl PCR master mix, 2 μl DNA and 2 μl barcode primers (2 μM equimolar mix of index 1 and index 2, Additional file 1) were mixed in a 384 well plate using a Tecan EVO robotic station (Tecan, Männedorf, Switzerland). 4.5 μl of the mix were transferred to the sample inlets of a primed 48.48 Fluidigm Access Array by the Tecan EVO. 4 μl target specific primers (1 μM in 1×AA loading reagent) were transferred to the primer inlets. Target specific and index primer were obtained from metabion (metabion international AG, Martinsread, Germany).

After loading, the chip was placed in an IFC Controller AX (Fluidigm Corporation, South San Fransisco, USA) for loading the PCR reagents before the PCR was performed on a FC1 cycler. Thermal profile was 50°C for 2 min, 70°C for 20 min, 95°C for 10 min, followed by 32 cycles at 95°C for 25 s, 60°C for 30 s and 72°C for 90 s, and a finishing step at 72°C for 5 min. The PCR products were harvested from the PCR chambers by an IFC Controller AX (Fluidigm Corporation, South San Francisco, USA).

### Dual indexing primers

Please refer to the supplementary information for the concept, indices and sample sheet information.

### Amplicon pooling, purification and quantification

The 48 samples of an Access Array chips were pooled. 8 pools were purified in parallel using AMPure XP (Beckman Coulter, Brea, USA) with a ratio of 0.7:1 beads to DNA. Purified amplicon pools were diluted 1:4000 for quantification by qPCR using an ECO Real-Time PCR cycler (Illumina, San Diego, USA) and the Library Quant Illumina Kit (KAPA Biosystems, Boston, USA) with standards in a range from 0.2 fM to 20 pM. Pooling, purification and dilution for quantification were performed on a Biomek 3000 workstation (Beckman Coulter, Brea, USA).

### Library preparation and MiSeq run

8 purified and quantified amplicon pools were mixed in equimolar amounts. The library was prepared as recommended by Illumina (MiSeq Reagent Kit v2 – Reagent Preparation Guide) and was loaded at 7.5 pM on one MiSeq flowcell with 10% phiX spiked in. Paired end sequencing was performed with 251 cycles.

Prior to the update of Real-Time Analysis Software (Illumina, San Diego, USA) to version 2.2.0.2, hardcoded matrix and phasing values were used for sequencing runs.

### Classification of reads for primer optimization

Primer sets to be tested were combined with samples of known HLA types on the Fluidigm chip and processed as described above. To assess the quality of the primer sets, the resulting MiSeq reads were classified in two ways: 1) Finding the most similar HLA allele(s) amongst all known HLA alleles by simple 10mer comparison. Up to 10 mismatches were allowed when considering the read as “matched” to a reference allele. Reads with more than 10 mismatches were classified as “not matched”. 2) Base by base comparison of reads with all known HLA alleles. The classification was based on the *Q* values of the mismatches. If the sum of the *Q* values of non-matching bases (*Q*_sum_) was >80, the read was regarded as “not matching”. If the read matched considerably worse (*Q*_sum_ difference >50) to the reference allele than to an allele as determined by approach 1, the read was regarded as “other HLA allele”. If the read matched to both alleles on the basis of the above criteria, the read was assigned to the allele with the lower *Q*_sum_. Reads fulfilling the matching criteria were tested to determine whether existing mismatches could be explained by PCR crossover, i.e. artificial recombinations of the two alleles during PCR. Those reads were classified as “Allele *n* with crossover” if at least two bases with good Q value (>25) matched to the alternate allele. Depending on PCR conditions and primer set, a considerable portion of the reads could be identified as “crossover” products. Reads fulfilling the matching criteria were tested to determine whether existing mismatches could be explained by PCR crossover. Those reads were classified as “Allele *n* with crossover” if at least two bases with good Q value (>25) matched to the alternate allele. “Not matched” reads were tested base by base against allele1 or allele2. If matching criteria were met, those reads were classified as “crossover”. In order to assess allele balancing, we calculated the ratio of the two alleles based on the classification for each primer set.

### neXtype

neXtype is designed for HLA typing from raw data provided by the Illumina MiSeq system. The methods implemented within neXtype take advantage of the large number of reads per sequence and the associated quality values (*Q* values). The software is specifically adapted to a paired-end amplicon sequencing approach. NeXtype is implemented as an Oracle PL/SQL application and scalable to any number of CPU cores and Oracle instances.

In a pre-typing run, neXtype assembles the input data that is independent of each specific run, such as HLA reference sequences from the IMGT/HLA database and primer information. The typing run itself is organized into several consecutive steps: primer recognition, allele matching, result classification, exon combination, result rating, and user interaction.

### Primer recognition

Primer recognition is carried out for each MiSeq read to assign HLA locus and exon. Primer recognition involves three levels:

Firstly, a string comparison between primer library and read is carried out.

If no match can be found then the read is compared to known artifacts of the primer set collected in a look-up table.

**Figure 7:**
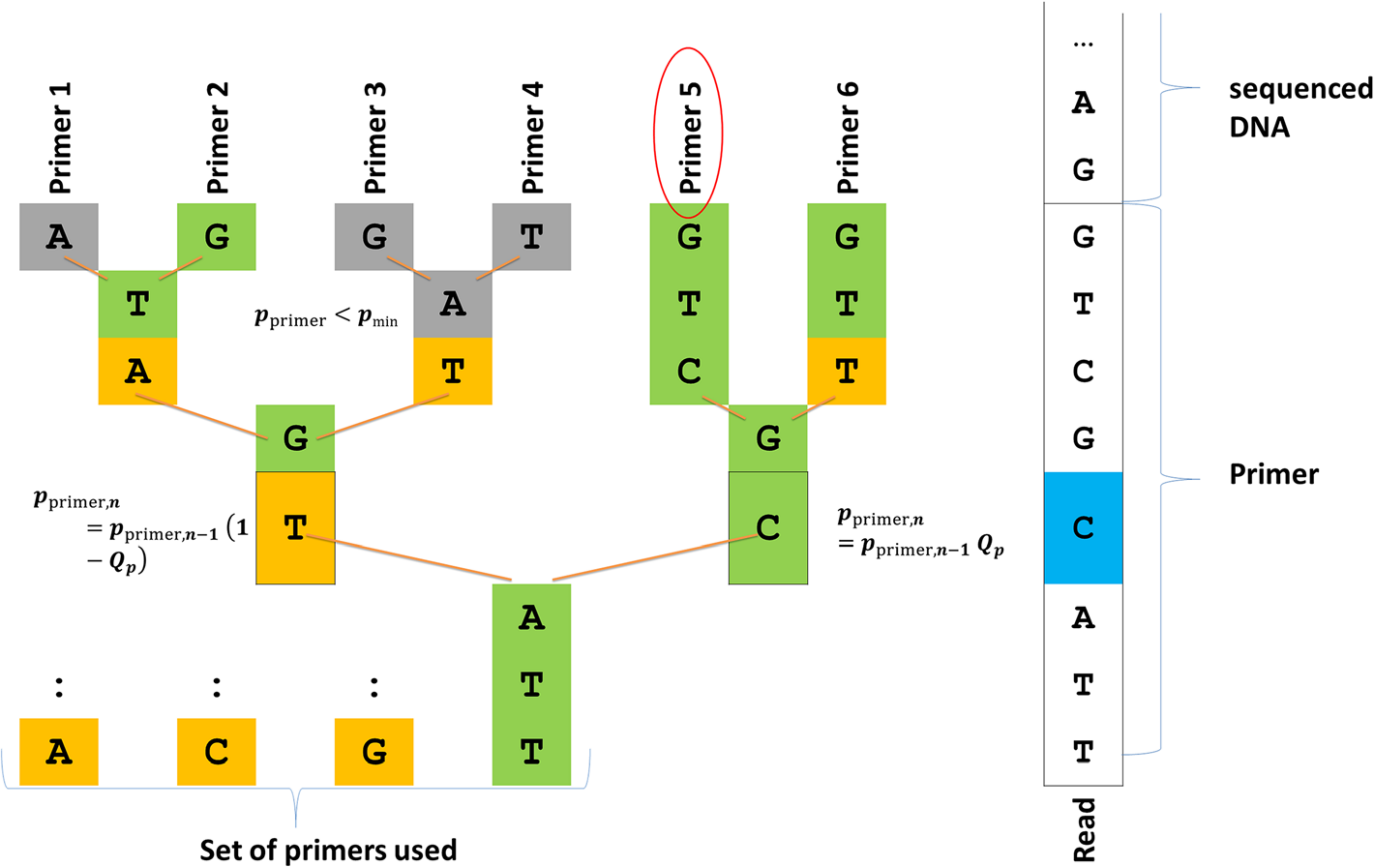
**Schematic representation of primer recognition level three.** In this example, primer 5 would have been assigned to the tested read.

If no match is found either, then on the third level the read is evaluated base-wise under consideration of *Q* values. For ease of calculation, we convert the *Q* values into numeric probabilities *Q*_*p*_ = 1-(1/(10^(*Q/10)*^). The primer sequence information is organized in a tree structure. At the beginning, all primers are considered to be equally probable matches for the given read and hence assigned a probability of *p*_primer_ = 1. Subsequently, each branch in the primer tree is compared with the base of the read sequence in a recursive manner. If the base at position *n* in the read matches a certain branch in the primer tree then that branch is followed and *p*_primer_ is multiplied by the respective *Q*_*p*_. If the branch does not match the primer base then that branch is followed, too, but *p*_primer_ is multiplied by (1-*Q*_*p*_). All branches are followed as long as *p*_primer_ does not drop below a predefined limit *p*_min_. When the read has been compared to each primer the process is stopped and the primer with the highest probability *p*_primer_ is retained as match if *p*_primer_ is sufficiently higher than the second best value (Figure 7). The sequence of the read that most closely matched a primer is stored in the primer artifacts look-up table for more rapid comparison of future reads.

### Allele matching

**Figure 8:**
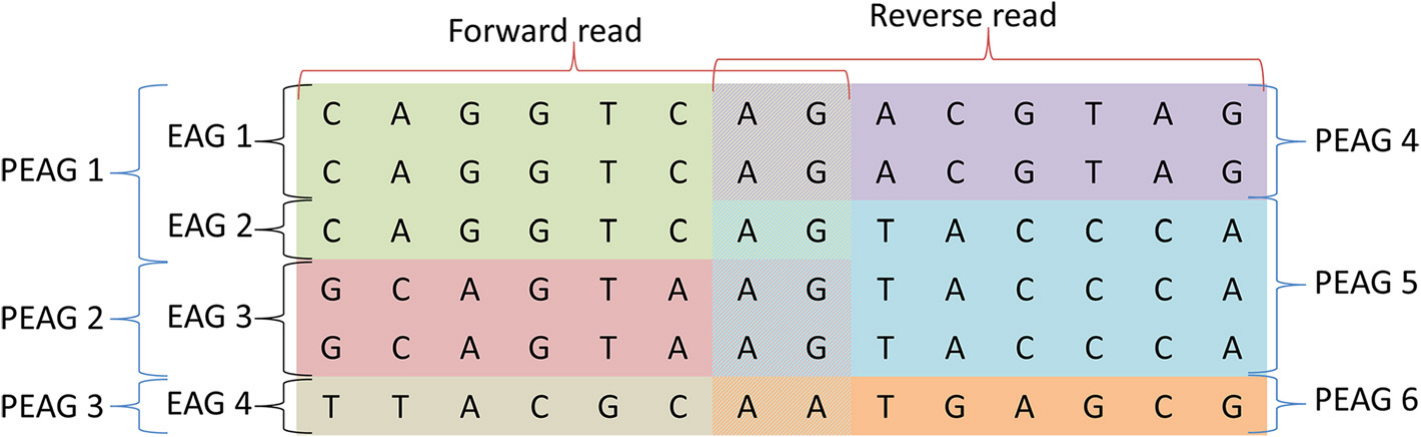
**Sketch showing generation of EAGs and PEAGs in forward and reverse read direction.** Each row represents the exon sequence of one HLA allele on a specific locus and exon. The chart shows how the EAGs and PEAGs in forward and reverse direction are generated.

Allele matching is carried out on exon level. Since various HLA alleles are identical over specific exons, we group such alleles into Exon Allele Groups (EAG). Matching of EAG is achieved by pre-assembling IMGT/HLA DNA database information in a tree structure under consideration of possible insertions and deletions in the introns. As MiSeq provides two reads for each typed exon, one in a forward and one in a reverse direction, and the read length is currently set to 251 bases, a value smaller than the typical exon length, the EAG are further grouped into partial EAG (PEAG) which include all alleles that have the same sequence in a given part of the exon for forward and reverse direction separately as determined by the read length. Each read is matched against all possible PEAG in a tree-structured manner just as in the third level of primer recognition. For each read, possible EAG are identified from the matched PEAG and associated with the respective probabilities *p*_EAG_ (Figure 8). Each EAG encompasses a total number of reads that are in principle similar enough to the EAG (“match”) which has the highest matching probability *p*_EAG_ – given the respective Qp values – to the EAG (“best match”) or, moreover, which is associated with only this specific EAG as “best match” (“single best match”). Only EAGs that score at least 5 “best match” reads are subsequently considered.

### Result classification

In typical cases, for each locus and exon more than two EAG are identified. NeXtype ranks these EAGs by score, i.e. number of “best match” reads in descending order. The possible classification of an EAG is as follows: result, potential new allele, noise, cross-over, and co-amplification. A base-by-base comparison of all “best match” reads to the EAG allows identification of potentially new alleles by identifying systematically occurring SNPs and evaluating the number of reads with this alteration. Likewise, the consensus sequence is determined based on the majority of base calls at each position. Using a binomial distribution, EAGs are checked pairwise to determine whether their occurrence can be explained as noise of higher scoring EAGs, given the number of reads and their *Q* values. EAGs are classified as “cross-over” if their consensus sequence can be obtained by combination of any two higher scoring EAGs. The consensus sequence is matched against the HLA system to identify off target PCR products (e.g. HLA-H, -DRB3, …). Those are marked as “co-amplificate”. EAGs not scoring at least 1/50 of the best scoring EAG are discarded. All EAGs not classified in the above categories are considered “results” by neXtype. If more than two EAGs are classified as “result”, neXtype indicates a warning or error depending on the relative number of “best matches” of the EAG.

### Exon combination

The final HLA assignments are obtained by combining the “results” from each exon. Only alleles that can be found in the two EAGs classified as “result” with the highest number of “best matches” in each exon become part of the final typing result. In addition to EAGs classified as “result”, EAGs classified as “co-amplificate” are considered if they are needed to generate a valid result. Exons with less than 20 reads in sum over all “result” EAGs are ignored. The collected alleles are coded into NMDP multi-allele or *G* codes [20,21]. In cases where results of exon 2 and exon 3 lead to more than one possible combination of exons (exon-shuffling), not all ambiguities can be resolved, thus resulting in intermediate resolution typing results.

### Result rating

To rate the quality of HLA assignments, neXtype uses several parameters, including the percentage of successful primer matches, the share of reads with a successful association to an EAG, and the percentage of reads in an exon which contribute to the final result in this specific exon. Each parameter has two predefined thresholds allowing classification of the HLA assignment’s quality. If a parameter exceeds the upper threshold, it scores 1, whereas if it falls below the lower threshold, it scores 0 and between the two thresholds the score is linearly interpolated between 0 and 1. The final rating is the product over all parameter scores. Furthermore, exons are checked for possible suppressed alleles using different thresholds depending on whether a homozygous or heterozygous result is retained.

### User interaction

All classified EAGs are displayed for each exon. The user is permitted to include or exclude EAGs from each exon based on his assessment and to recombine the exons for a final typing result. Any user interaction triggers recomputing of result ratings. The user can further mark individual EAGs as known artifacts that should be ignored in future runs. When the user agrees with the HLA assignment by neXtype, a status “finished” can be set and the result is submitted electronically. HLA assignments achieving an overall rating of 1 are automatically submitted.

## Abbreviations

HLA: Human leukocyte antigen; NGS: Next generation sequencing; SPRI: Solid phase reversible immobilization; GSSP: Group specific sequencing primer.

## Competing interests

The authors declare that they have no competing interests.

## Authors’ contributions

VL and IB designed the workflow and coordinated implementation. IB, KL, JMA and VA developed and optimized the primer sets. KL and VA performed the experiments. PP and JMA programmed the automation devices. JH, JS, JP, VL and AHS conceived the neXtype algorithms. JH, JS, JN and UE developed neXtype. VL, BS and DMB wrote custom scripts for data analysis. KL, BS, JMA, DMB and IB performed data analysis and validated the neXtype software. CS coordinated validation and routine operation. JP coordinated neXtype development. GE and AHS supervised the work. All authors read and approved the final manuscript.

## Supplementary Material

Additional file 1 **Sequence information for the dual indexing, 4-primer approach is available as part of an additional file.** Sequences include inner and outer primers, index sequences for index 1 (96 sequences) and index 2 (16 sequences) as well as a model sample sheet. Click here for file
